# Association Between Thoracic Kyphosis and Hiatal Enlargement: A CT-Based Study Interpreted in Light of GERD-Linked Morphological Markers

**DOI:** 10.3390/tomography11090098

**Published:** 2025-08-26

**Authors:** Mustafa Mazıcan, Ismail Karluka, Davut Tuney

**Affiliations:** 1 Department of Radiology, Division of Interventional Radiology, Başkent University Dr. Turgut Noyan Application and Research Center, 01250 Adana, Turkey; ismailkarluka@baskent.edu.tr; 2 Department of Radiology, Faculty of Medicine, Marmara University, 34722 Istanbul, Turkey; datuney@hotmail.com

**Keywords:** thoracic kyphosis, hiatal surface area, gastroesophageal reflux, computed tomography, diaphragmatic anatomy, Cobb angle

## Abstract

Background: Thoracic kyphosis has been increasingly associated with altered intra-abdominal and diaphragmatic dynamics, potentially contributing to gastroesophageal reflux disease (GERD) and hiatal hernia (HH). While previous studies have shown a relationship between spinal deformities and GERD symptoms, these findings have been largely observational, with few morphometric analyses. No prior study has directly quantified the relationship between thoracic curvature and hiatal surface area (HSA) using standardized computed tomography (CT)-based methods. Furthermore, existing studies have typically focused on patients with visible hernias, limiting understanding of early, subclinical anatomical changes. This study addresses this gap by evaluating whether thoracic kyphosis is associated with measurable hiatal enlargement, even in the absence of overt HH. Methods: In this retrospective, single-center study, 100 adult patients (50 with thoracic kyphosis, defined as a Cobb angle of ≥50° and 50 age- and sex-matched controls) underwent multidetector CT (MDCT). Hiatal surface area (HSA) was measured on a standardized oblique axial plane aligned with the diaphragmatic crura. Correlation and multivariable regression analyses were performed to assess relationships between Cobb angle and HSA. Results: The kyphosis group showed significantly larger HSA than controls (5.14 ± 1.31 cm^2^ vs. 3.59 ± 0.74 cm^2^; *p* < 0.001). A moderate positive correlation was found between Cobb angle and HSA (r = 0.336, *p* = 0.017). Multivariable analysis identified the Cobb angle as an independent predictor of HSA (β = 0.028; *p* = 0.017), while age and sex were not significant predictors. No overt herniation was present in any subject. Conclusions: This is the first CT-based morphometric study to demonstrate that thoracic kyphosis is associated with hiatal enlargement, even in the absence of overt herniation. These findings support the hypothesis that postural spinal deformities may predispose individuals to GERD by structurally remodeling the diaphragmatic hiatus.

## 1. Introduction

Thoracic kyphosis refers to an excessive anterior–posterior curvature of the thoracic spine, most commonly assessed using the Cobb method on sagittal images. Although some degree of kyphosis is a regular part of the spine’s sagittal alignment—typically ranging between 20° and 40°—a marked increase beyond this range may result in structural and functional consequences [1]. The kyphotic curve may progress due to a range of etiological factors, including trauma, congenital vertebral anomalies, Scheuermann disease, infections, degenerative disc disease, and post-surgical iatrogenic changes [1]. Importantly, thoracic kyphosis tends to increase with age and can be more pronounced in females during specific growth periods, though it is overall more prevalent in males [1]. In addition to age-related structural changes, thoracic kyphosis may also be influenced by sex-specific differences in muscle strength and postural control. In a study focusing on adults aged 35–45, women were found to exhibit significantly greater thoracic curvature than men, reflecting biomechanical and neuromuscular differences between sexes [2].

Kyphosis, particularly in the context of osteoporotic vertebral fractures, has been increasingly recognized as a contributor to altered intra-abdominal dynamics, potentially predisposing individuals to gastroesophageal reflux disease (GERD) and hiatal hernia. This association is particularly evident in patients with lumbar kyphosis, where deformity-related increases in intra-abdominal pressure may exert mechanical stress on the gastroesophageal junction [3].

The herniation of gastric contents characterizes hiatal hernia (HH) through the esophageal hiatus of the diaphragm and is a well-established anatomical contributor to GERD. The biomechanical interplay between thoracic spinal curvature and abdominal pressure gradients is hypothesized to cause expansion of the hiatus, facilitating upward migration of the stomach [4,5]. This hypothesis gains support from several studies associating spinal deformities such as kyphosis and scoliosis with increased prevalence of HH and reflux symptoms [6,7].

Sagittal reconstructed MDCT images enable the measurement of thoracic kyphosis by providing a clear visualization of vertebral alignment from T1 to T12. This method provides a noninvasive and practical approach to assessing spinal curvature in preoperative evaluations [8]. Additionally, MDCT provides accurate quantification of the hiatal surface area and esophageal hiatus diameters, including anterior–posterior and transverse dimensions, which are valuable in the preoperative evaluation of gastroesophageal reflux disease and hiatal hernia [9].

In a key study, Karatay et al. [9] utilized MDCT to evaluate the HSA and esophageal hiatus diameters, reporting significantly increased values in patients with GERD compared to controls. Their findings validated CT as a diagnostic tool and established a morphometric basis for the link between anatomical deformation and functional impairment. This perspective is further supported by Ouyang et al. [10], who introduced a standardized method for in vivo quantification of the esophageal hiatus using double-oblique multiplanar reconstructed MDCT images. They demonstrated that HSA was significantly larger in patients with HH, particularly those with type III hernias, and also correlated with the presence of GERD. These results reinforce the clinical relevance of anatomical assessment in understanding reflux pathophysiology and highlight the potential of MDCT-based measurements to guide preoperative planning in antireflux surgery [10].

Vertebral deformities such as thoracic kyphosis and degenerative scoliosis have been shown to alter intra-abdominal dynamics and diaphragmatic tension, contributing to the pathogenesis of GERD and hiatal hernia. Kusano et al. demonstrated a significant correlation between the severity of kyphosis and the grade of hiatal hernia in older women, suggesting that kyphotic angulation may stretch the esophagus cranially and widen the diaphragmatic hiatus independent of obesity [5]. Similarly, Hosogane et al. identified a strong association between left convex lumbar scoliosis and GERD symptoms, hypothesizing that spinal curvature near the diaphragm may distort the esophageal hiatus and increase intra-abdominal pressure [7]. Batirel et al. further emphasized that even small increases in intra-abdominal pressure can overcome reduced lower esophageal sphincter (LES) pressure in the setting of a large esophageal hiatus, especially in individuals with compromised crural support, leading to increased acid reflux and esophagitis [11]. These findings suggest that spinal deformities may create a biomechanical environment that favors the development of hiatal hernia and GERD, particularly in elderly populations.

Building on these observations, we hypothesize that increased thoracic kyphosis may be associated with measurable changes in the morphology of the esophageal hiatus, particularly an increase in hiatal surface area (HSA), which may predispose patients to GERD and HH. Despite the established roles of spinal deformity and hiatus dilation in reflux pathophysiology, this interaction remains underexplored with direct morphometric methods, and no prior study has directly evaluated the anatomical relationship between thoracic kyphosis and hiatus morphology using quantitative imaging. By exploring this association through sagittally reconstructed MDCT, this study aims to provide novel insights into the mechanical impact of thoracic curvature on the diaphragmatic hiatus. Understanding this relationship may enhance preoperative risk stratification, aid in the early identification of anatomical susceptibility to GERD, and ultimately contribute to more personalized and anatomy-informed surgical planning in affected populations.

## 2. Materials and Methods

### 2.1. Study Design and Ethical Approval

This single-center, retrospective observational study was performed at Marmara University School of Medicine, Department of Radiology. The study period spanned 1 April 2012 through 30 April 2013. The Institutional Review Board of Marmara University approved the protocol (Approval No. 09.2013.0089; dated 2 April 2013). Prior to imaging, all patients signed a written informed consent form titled *Patient Information and Consent Form for Computed Tomography Examination*.

### 2.2. Participants

A total of 100 adult patients (50 with thoracic kyphosis and 50 controls) were included in this study. The age range was 25–90 years (mean 56.47 ± 15.49 years). There were a total of 45 females and 55 males. Group allocation was based on Cobb angle measurements as described below.

The study population consisted of adults who underwent thoracic CT scans for various clinical indications and had no prior history of spinal or upper gastrointestinal surgery. As such, the sample may be considered representative of a general adult population undergoing diagnostic thoracic imaging in tertiary care settings.

### 2.3. Patient Selection and Group Allocation

We screened 500 consecutive thoracic CT examinations acquired for any clinical indication during the study window. From this pool, we identified 50 patients meeting the criteria for thoracic kyphosis (Cobb angle ≥ 50°) and randomly selected 50 age- and sex-matched control subjects with Cobb angles between 20° and 40°.

The inclusion criteria comprised adults aged 18 years or older who had available high-resolution thoracic CT data, either contrast-enhanced or non-contrast, obtained on a 256-slice scanner, and no prior history of upper gastrointestinal or spinal surgery. Patients were excluded if they had radiographic evidence of hiatal hernia or diaphragmatic pathology, large intrathoracic or intra-abdominal masses (such as tumors or significant ascites) that could distort hiatus anatomy, or poor image quality due to motion or beam-hardening artifacts. Additional exclusion criteria were the presence of connective tissue disorders (e.g., Marfan syndrome) or systemic diseases affecting diaphragmatic integrity, prior spinal instrumentation at vertebral levels T4–T12, and marked obesity (BMI ≥ 30 kg/m^2^), given its known influence on intra-abdominal pressure and hiatus morphology [12].

### 2.4. CT Acquisition Protocol

All scans were obtained on a SOMATOM Definition Flash 256-slice MDCT scanner (Siemens, Erlangen, Germany) using the following parameters:

- Tube voltage: 120 kVp;
- Tube current: 180–250 mA with automatic exposure control;
- Detector collimation: 128 × 0.6 mm;
- Rotation time: 0.5 s;
- Slice thickness: 5 mm with 1 mm overlap for multiplanar reformations.

Both contrast-enhanced and non-contrast thoracic CT examinations were retrospectively included, depending on clinical indication at the time of acquisition.

Contrast-enhanced studies used 80–100 mL of nonionic iodinated contrast (350 mg I/mL) administered at a rate of 3.5 mL/s, followed by a 30 mL saline flush. Images were reconstructed in axial, sagittal, and coronal planes using a standard soft-tissue kernel.

### 2.5. Image Post-Processing and Standardization

All CT datasets were transferred to a dedicated workstation (Syngo MMWP VE40A, Siemens, Germany) for multiplanar reconstruction and analysis. A standardized image post-processing protocol was implemented to ensure reproducibility and minimize measurement variability across patients.

First, sagittal and coronal thoracic planes were used as anatomical references to define a doubly oblique axial plane. The image was realigned to bisect the midline of the vertebral bodies in the sagittal view and to run parallel to the anterior vertebral surfaces in the coronal view. The final oblique axial plane was then defined as perpendicular to the sagittal axis and aligned with the diaphragmatic crura, providing the optimal cross-sectional view of the esophageal hiatus.

**Figure 1:**
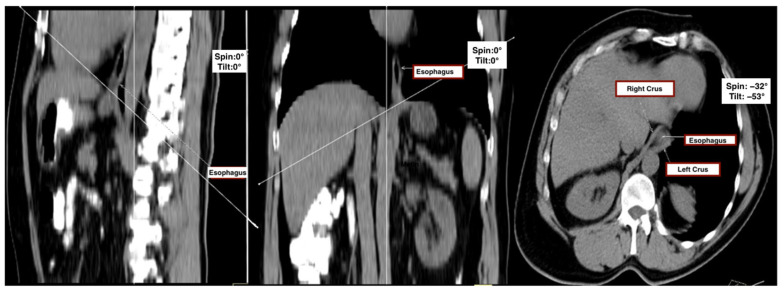
Multiplanar CT evaluation of the esophageal hiatus. Standardized sagittal (**left**), coronal (**middle**), and oblique axial (**right**) MDCT reconstructions used for anatomical localization and morphometric analysis of the esophageal hiatus. The esophagus and diaphragmatic crura (right and left) are labeled. The oblique axial plane was obtained by aligning with the diaphragmatic crura and orthogonal to the sagittal axis to best visualize the hiatal components for surface area measurement.

This plane, which corresponded closely to intraoperative anatomical appearance and enabled complete visualization of hiatal components, was accepted as the reference plane for all measurements. To assess consistency across patients, the tilt angle (sagittal deviation) and spin angle (coronal deviation) were recorded for each subject (Figure 1).

Hiatal surface area (HSA) was quantified on this standardized plane using a freehand region-of-interest (ROI) tool. The inner margin of the esophageal hiatus—bounded by the crural limbs—was manually traced to enclose the whole cross-sectional opening. In cases where fat planes were indistinct, the anterior aortic contour or adjacent esophageal border was used as an anatomical reference. The workstation software automatically calculated the enclosed area in square centimeters. All reconstructions were reviewed using uniform soft-tissue window settings (width: 400 HU, level: 40 HU) to ensure consistency in soft-tissue contrast and anatomical margin delineation.

### 2.6. Assessment of Thoracic Kyphosis

Thoracic kyphosis was quantitatively assessed using the standard Cobb method, as recommended by the Scoliosis Research Society [13]. On sagittal reformatted CT images, the Cobb angle was measured between the superior endplate of the T1 vertebra and the inferior endplate of the T12 vertebra. Two lines were drawn parallel to these respective endplates, and perpendicular lines were extended from each. The angle formed at the intersection of these perpendiculars represented the thoracic kyphosis angle.

In this study, patients with a Cobb angle between 20° and 40° were classified as the control group, while those with a Cobb angle of 50° or greater were assigned to the kyphosis group.

This method provides a reproducible and widely accepted measure of sagittal spinal curvature and is particularly well-suited for use in thoracic CT datasets.

### 2.7. Sample Size Justification

Sample size calculation was performed using PASS 2008 (NCSS, LLC, Kaysville, UT, USA) and based on preliminary data suggesting a mean difference of 1.5 cm^2^ in hiatal surface area (HSA) between kyphotic and control groups, with a pooled standard deviation of 2.0 cm^2^, a two-tailed *t*-test with α = 0.05 and power (1 − β) = 0.80 indicated that 45 subjects per group would be required to detect a statistically significant difference. To account for potential exclusions and missing data, 50 subjects were included per group.

### 2.8. Statistical Analysis

All statistical analyses were conducted using NCSS 2007 and PASS 2008 software (Number Cruncher Statistical System, Kaysville, UT, USA). Continuous variables were expressed as mean ± standard deviation, and categorical variables were expressed as counts and percentages.

Group comparisons for continuous variables (e.g., age, Cobb angle, hiatal surface area, spin and tilt angles) were performed using the independent samples Student’s *t*-test. Categorical variables (e.g., sex distribution) were compared using the chi-square test with Yates correction when appropriate.

The relationships between continuous variables were assessed using Pearson correlation coefficients. To identify independent predictors of hiatal surface area (HSA), a multivariable linear regression analysis was performed, including Cobb angle, age, and sex as covariates. The strength of the model was reported using the coefficient of determination (R^2^).

Although no formal normality test was applied, the approximately symmetric distributions observed during visual inspection, along with the relatively large and balanced group sizes (n = 50 per group), were deemed acceptable for the application of parametric tests.

A two-sided *p*-value of < 0.05 was considered statistically significant throughout the analysis.

## 3. Results

A total of 100 patients (50 with kyphosis and 50 controls) were included in this study. Ages ranged from 25 to 90 years (mean 56.47 ± 15.49 years); 45 (45%) were female and 55 (55%) were male.

Demographic Comparison

- Age: The kyphosis group was significantly older than controls (65.62 ± 11.45 vs. 47.32 ± 23.51 years, *p* < 0.01);
- Sex: There was no significant difference in sex distribution between groups (kyphosis: 22 F/28 M; control: 23 F/27 M; *p* = 1.00) (Table 1).

**Table 1:** Demographic and measurement comparisons by group.

Variable	Kyphosis Group (n = 50)	Control Group (n = 50)	*p*-Value
Age (years)	65.62 ± 11.45	47.32 ± 23.51	0.001 **
Gender			1.000
Female, n (%)	22 (44%)	23 (46%)	
Male, n (%)	28 (56%)	27 (54%)	
Cobb Angle (°)	57.14 ± 7.64	32.64 ± 5.85	0.001 **
Hiatus Surface Area (cm^2^)	5.14 ± 1.31	3.59 ± 0.74	0.001 **
Spin Angle (°)	36.18 ± 9.47	40.66 ± 12.06	0.041 *
Tilt Angle (°)	38.88 ± 7.46	41.94 ± 8.36	0.035 *

Values are presented as mean ± standard deviation or n (%).* *p* < 0.05; ** *p* < 0.01. Note: Age and Cobb angle differences between groups indicate significant demographic and structural differences that affect hiatal measurements.

Spinal and Hiatal Measurements

**Figure 2:**
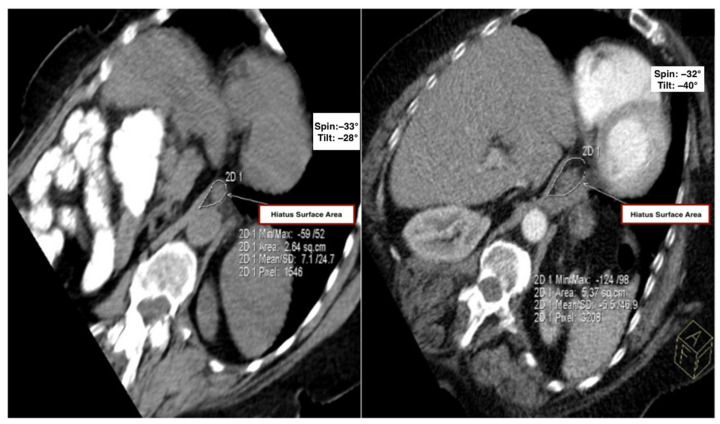
Comparison of the hiatal surface area between the control and kyphosis groups. Representative oblique axial MDCT images from the control group (**left**) and the kyphosis group (**right**), demonstrating measurement of hiatal surface area using a freehand region-of-interest (ROI) tool.

- Cobb Angle: As expected, the mean Cobb angle was higher in the kyphosis group (57.14 ± 7.64°) versus controls (32.64 ± 5.85°, *p* < 0.001);
- Hiatal Surface Area (HSA): Mean HSA was significantly larger in the kyphosis group compared to controls (5.14 ± 1.31 cm^2^ vs. 3.59 ± 0.74 cm^2^; *p* < 0.001). A mean difference of 1.55 cm^2^ in HSA may appear modest, yet it represents a greater than 40% relative increase compared to controls, indicating substantial structural remodeling of the diaphragmatic hiatus that may facilitate gastric content migration and contribute to early reflux pathophysiology (Table 1 and Figure 2).

- Spin and Tilt Angles

Measurements of “spin” and “tilt” (angular deviation from actual coronal and sagittal planes) were significantly lower in the kyphosis group compared to controls (*p* < 0.05), suggesting that altered vertebral orientation contributes to hiatal remodeling (Table 1).

Group-Specific Hiatal Associations

- Kyphosis Group: No significant differences in HSA by sex (females: 5.11 ± 1.54 cm^2^ vs. males: 5.17 ± 1.14 cm^2^; *p* = 0.878), and no age–HSA correlation (r = 0.073, *p* = 0.616);
- Control Group: Male controls had larger HSA than females (3.79 ± 0.74 cm^2^ vs. 2.36 ± 0.68 cm^2^; *p* = 0.038), while age was not correlated with HSA (r = 0.250, *p* = 0.078) (Table 2).

**Table 2:** Relationships between hiatal surface area and demographic/clinical variables.

Analysis	Group	Value (Mean ± SD or r)	*p*-Value
Hiatus Surface Area by Gender (cm^2^)	Kyphosis	Female: 5.11 ± 1.54; Male: 5.17 ± 1.14	0.878
	Control	Female: 2.36 ± 0.68; Male: 3.79 ± 0.74	0.038 *
Correlation: Hiatus Surface Area vs. Age (r)	Kyphosis	r = 0.073	0.616
	Control	r = 0.250	0.078
Correlation: Hiatus Surface Area vs. Cobb Angle (r)	Kyphosis	r = 0.336	0.017 *
	Control	r = 0.035	0.808

In the kyphosis group, gender did not significantly affect hiatus surface area; in controls, males had larger hiatal areas. Only the kyphosis group showed a significant correlation between Cobb angle and hiatal opening. * *p* < 0.05. r: Pearson correlation coefficient, indicating the strength and direction of a linear relationship between two continuous variables.

Correlation Between Cobb Angle and HSA

- In the kyphosis group, Cobb angle and HSA showed a moderate positive correlation (r = 0.336, *p* = 0.017), indicating that greater spinal curvature was associated with a larger hiatal area;
- In controls, no significant correlation was observed (r = 0.035, *p* = 0.808) (Table 2).

Multivariable Regression Analysis: Independent Predictors of Hiatal Surface Area

A multivariable linear regression model was fitted to identify independent predictors of hiatal surface area (HSA). As summarized in Table 3, Cobb angle was the sole statistically significant predictor (β = 0.028 cm^2^/°, 95% CI 0.005–0.051; *p* = 0.017), indicating that each one-degree increase in thoracic kyphosis corresponded to a 0.028 cm^2^ increase in HSA. Neither age (β = 0.004 cm^2^ per year, 95% CI −0.003–0.010; *p* = 0.250) nor sex (male vs. female; β = 0.35 cm^2^, 95% CI −0.40–1.10; *p* = 0.360) reached statistical significance. The overall model explained 11.3% of the variance in HSA (R^2^ = 0.113), a relatively modest proportion that nevertheless underscores a clinically relevant contribution of spinal curvature to hiatal morphology.

## 4. Discussion

This study retrospectively investigated the effect of thoracic kyphosis on the hiatal surface area (HSA) using multidetector computed tomography (MDCT), revealing a significantly larger HSA in the kyphotic group compared to the control group. A moderate positive correlation between the Cobb angle and HSA was observed, indicating that spinal deformity may structurally alter the diaphragmatic hiatus. Although the dataset originates from 2013, its scientific relevance persists and aligns closely with recent findings published between 2015 and 2025.

Several studies have emphasized the impact of kyphosis on intra-abdominal pressure and diaphragmatic displacement. Talarico and Vlahu [14] demonstrated that spinal deformities, such as kyphoscoliosis, can anatomically stretch and widen the esophageal hiatus, facilitating intrathoracic stomach migration and the development of HH. Masaoka et al. [15] demonstrated that enlargement of the anterior–posterior thoracic diameter—reflected by a decreased Haller Index—is associated with hiatal hernia in elderly patients, suggesting that thoracic deformity may play a structural role in hiatal widening. Building on these findings, Polomsky et al. [4] provided further evidence by showing that patients with intrathoracic stomachs had significantly greater thoracic kyphosis—as measured by Cobb angle—and more vertebral fractures compared to age- and gender-matched controls. Their data indicated a mean Cobb angle of 50.2° in patients with HH, significantly higher than the 39.7° observed in controls (*p* < 0.001), particularly among female patients. These findings strengthen the hypothesis that spinal curvature may not only distort diaphragmatic geometry but also contribute mechanically to the superior migration of abdominal viscera, reinforcing the role of thoracic biomechanics in the pathogenesis of HH. These previous insights are echoed in our current study, where we observed a significantly larger hiatal surface area (HSA) in patients with pronounced thoracic kyphosis (Cobb angle ≥ 50°) compared to the control group. More importantly, the moderate positive correlation identified between Cobb angle and HSA (r = 0.336, *p* = 0.017) supports the notion that spinal curvature may not only distort the diaphragmatic architecture but also actively contribute to the superior displacement of abdominal contents. This direct morphometric evidence strengthens the hypothesis that thoracic kyphosis plays a biomechanical role in the pathogenesis of HH, bridging the anatomical observations of prior cadaveric and clinical studies with objective radiological data.

Karatay et al. [9] demonstrated that computed tomography-based measurement of hiatal surface area (HSA) is a reliable method, reporting significantly larger HSA values in GERD patients compared to controls (median 19.11 cm^2^ vs. 4.89 cm^2^; *p* < 0.001) and highlighting its utility in distinguishing hiatal hernia (HH) subtypes. Moten et al. [16] validated a multiplanar MDCT technique for measuring HSA against intraoperative findings, demonstrating a strong correlation (r = 0.83; *p* < 0.001) and excellent intra- and inter-observer agreement (ICC = 0.97), confirming its reproducibility. Chan et al. [17] further supported this approach, demonstrating that CT-HSA measurements not only significantly differentiated HH patients from matched controls (8.7 cm^2^ vs. 2.2 cm^2^; *p* < 0.01) but also yielded a sensitivity of 94.6% and an ICC of 1.00, outperforming traditional modalities such as endoscopy, barium swallow, and HRM. Similarly, Ouyang et al. [10] found that patients with HH had significantly larger HSA values (mean 6.9 cm^2^) compared to controls (2.5 cm^2^; *p* < 0.0001), with type III hernias showing the most significant enlargement. However, in patients without HH, HSA was not predictive of GERD (3.0 cm^2^ vs. 2.5 cm^2^; *p* = 0.12). Shi et al. [18] extended these findings by demonstrating that, even in the absence of radiologically visible HH, increased oesophageal hiatus surface area (OHSA) measured via MDCT correlated positively with acid exposure time (r = 0.47; *p* < 0.001) independent of lower esophageal sphincter pressure. Our study aligns substantially with these findings. We found that patients with thoracic kyphosis had significantly larger HSA values compared to controls (5.14 ± 1.31 cm^2^ vs. 3.59 ± 0.74 cm^2^; *p* < 0.001), and Cobb angle correlated moderately with HSA (r = 0.336; *p* = 0.017). By demonstrating this association in a morphologically intact population—excluding patients with visible hernias—our results support the growing evidence that HSA enlargement may represent a preclinical anatomical marker of GERD susceptibility. Although the explained variance was modest (R^2^ = 0.113), the significance of the Cobb angle as an independent predictor highlights the anatomical influence of thoracic curvature, while also suggesting that additional biomechanical or physiological factors may contribute to HSA variation. Thus, CT-derived HSA should be recognized not only as a diagnostic endpoint but also as a sensitive and objective biomarker for early risk stratification in patients with posture-related anatomical alterations such as thoracic kyphosis. Given its reproducibility and predictive potential, CT-based HSA measurement may serve as a noninvasive anatomical biomarker to identify GERD susceptibility in postural deformities even before symptomatic reflux or herniation occurs.

Although MRI can provide excellent soft-tissue contrast, CT remains the most validated and widely used modality for hiatal morphometry, with strong reproducibility and correlation to intraoperative findings [9,10,16,17]. By contrast, MRI has been less frequently applied in this context, and its role in routine clinical practice remains limited.

These findings are further contextualized by the normative data established by Dass et al. [19], who retrospectively evaluated the esophageal hiatal surface area (HSA) in 119 healthy adults using multiplanar MDCT reconstructions. They reported a mean HSA of 2.88 cm^2^ in males and 2.51 cm^2^ in females, with an overall mean of 2.70 cm^2^ (range: 1.3–5.4 cm^2^). They found that sex, but not age, was significantly associated with HSA. Notably, their study excluded individuals with obesity, visible hernias, or diaphragmatic abnormalities, establishing a physiologic reference range for normal hiatus dimensions. When these reference values are applied to our cohort, the significantly larger HSA observed in the kyphosis group (mean 5.14 ± 1.31 cm^2^) exceeds the expected normal range even among males, suggesting that thoracic spinal curvature may exert a structural influence on diaphragmatic anatomy beyond physiologic variation. The absence of age-related HSA changes in both the Dass et al. [19] study and our control group further reinforces the interpretation that kyphosis, rather than age, underlies the observed hiatal remodeling. Thus, our results align with previously validated CT morphometry techniques and suggest that postural deformities may be associated with subclinical anatomical changes in the esophageal hiatus. These changes could potentially contribute to GERD and HH, but further prospective studies are necessary to confirm this relationship.

## 5. Limitations

This study has several limitations. First, the multivariable regression model explained only 11.3% of the variance in hiatal surface area (R^2^ = 0.113), indicating that the majority of HSA variability remains unaccounted for by the Cobb angle, age, and sex. Factors such as diaphragmatic muscle tone, body habitus, or subclinical herniation may also influence hiatus morphology and warrant investigation in future work.

Second, by design, we excluded all patients with overt hiatal hernias or other diaphragmatic pathologies. This selection criterion likely narrowed the observed range of HSA values, limiting the generalizability of our findings to patients who already exhibit herniation. Prospective cohorts that encompass the full spectrum of hiatal anatomy are needed to determine whether kyphosis–HSA relationships hold true in these populations.

Third, although age did not emerge as a significant predictor in our model, the inclusion of adults spanning 25 to 90 years may mask age-specific effects on HSA. Future studies should consider stratified analyses or narrower age cohorts to explore potential interactions between aging and spinal curvature on hiatus remodeling.

Fourth, the retrospective, single-center nature of this investigation, along with its modest sample size, may limit the external validity of our results. Larger, multicenter prospective studies will be essential to confirm these preliminary observations and to assess additional clinical and anatomical covariates.

Finally, a key limitation of our study is the absence of long-term follow-up data to evaluate whether patients with hiatal enlargement subsequently developed overt herniation. This lack of longitudinal data prevents us from assessing the predictive value of CT-based hiatus measurements for future herniation. Future prospective studies with long-term surveillance are required to determine whether hiatal widening in patients with thoracic kyphosis may serve as an early predictor of clinically significant hiatal hernia.

## 6. Conclusions

This study provides the first CT-based morphometric evidence that thoracic kyphosis is associated with measurable enlargement of the esophageal hiatus, even in the absence of overt herniation. A statistically significant increase in hiatal surface area (HSA) was observed in individuals with kyphotic deformity, with the Cobb angle emerging as an independent anatomical predictor. Although the explained variance was modest, these findings support the hypothesis that spinal curvature contributes to early structural remodeling of the diaphragmatic hiatus. Given its reproducibility, CT-derived HSA measurement may contribute to anatomical risk stratification in patients with postural spinal deformities. These findings suggest a possible role for CT-based morphometry in identifying anatomically at-risk populations, although further prospective studies are required to establish predictive value.

## Figures and Tables

**Table 3:** Multivariable regression analysis of hiatal surface area predictors.

Analysis	Group	Value (β [95% CI])	*p*-Value
Multivariable regression	Cobb angle	0.028 (0.005–0.051)	0.017 *
	Age	0.004 (−0.003–0.010)	0.250
	Sex (male = 1)	0.35 (−0.40–1.10)	0.360
Model evaluation	—	R^2^ = 0.113	—

Note: β, regression coefficient; CI, confidence interval; * *p* < 0.05 indicates statistical significance. The model R^2^ reflects the proportion of variance in the hiatal surface area explained by the model.

## Data Availability

The data sets utilized and/or analyzed during the current study are available from the corresponding author upon reasonable request.
